# Correction: Cultures of Trust: Effects of Avatar Faces and Reputation Scores on German and Arab Players in an Online Trust-Game

**DOI:** 10.1371/journal.pone.0107075

**Published:** 2014-08-25

**Authors:** The PLOS ONE Staff

## Notice of Republication

This article was republished on August 7, 2014, to replace incorrectly changed characters in the byline and citation. The publisher apologizes for the errors. Please download this article again to view the correct version. The originally published, uncorrected article and the republished, corrected article are provided here for reference.

## Supporting Information

File S1 Originally published, uncorrected article.
(PDF)

File S2 Republished, corrected article.
(PDF)
