# Supplementary material for: Correction: Cultures of Trust: Effects of Avatar Faces and Reputation Scores on German and Arab Players in an Online Trust-Game
Source: PLoS One. 2014 Aug 25;9(8):e107075. doi: 10.1371/journal.pone.0107075 (PMC4143361; doi:10.1371/journal.pone.0107075)
Supplement: File S1 — Originally published, uncorrected article. (PDF) [file pone.0107075.s001.pdf]

OPEN ACCESS Freely available online

PLOS | ONE

Cultures of Trust: Effects of Avatar Faces and Reputation Scores on German and Arab Players in an Online Trust-Game

Gary Bente[1], Thomas Dratsch[1]*, Kai Kaspar[2], Tabea Häβler[1], Oliver Bungard[1], Ahmad Al-Issa[3]

1 Communication and Media Psychology, Department of Psychology, University of Cologne, Cologne, Germany, **2** Social and Media Psychology, Department of Psychology, University of Cologne, Cologne, Germany, **3** Department of English, American University of Sharjah, Sharjah, United Arab Emirates

Abstract

Reputation systems as well as seller depictions (photos; avatars) have been shown to reduce buyer uncertainty and to foster trust in online trading. With the emergence of globalized e-markets, it remains an urgent question whether these mechanisms, found to be effective for Western cultures, also apply to other cultures. Hypothesizing that members of collectivistic cultures in contrast to those of individualistic cultures would rely more on visual social cues (seller faces) than on factual information (reputation scores), we compared buying decisions of Arab and German participants in an experimental trust game. Photo-realistic avatars were used instead of photos to control facial features and expressions. The results revealed significant main effects for both reputation scores and avatar faces. Moreover, both variables significantly affected the purchase behavior of Arab as well as German buyers, suggesting cross-cultural universals in the processing of trust cues. The results have implications for future cross-cultural studies in e-commerce as well as the design of online markets and shared virtual environments.

Citation: Bente G, Dratsch T, Kaspar K, Häβler T, Bungard O, et al. (2014) Cultures of Trust: Effects of Avatar Faces and Reputation Scores on German and Arab Players in an Online Trust-Game. PLoS ONE 9(6): e98297. doi:10.1371/journal.pone.0098297

Editor: Frank Krueger, George Mason University/Krasnow Institute for Advanced Study, United States of America

Received June 24, 2013; **Accepted** April 30, 2014; **Published** June 5, 2014

Copyright: © 2014 Bente et al. This is an open-access article distributed under the terms of the Creative Commons Attribution License, which permits unrestricted use, distribution, and reproduction in any medium, provided the original author and source are credited.

Funding: This study was supported by a project grant of the VW Foundation to Gary Bente. The funders had no role in study design, data collection and analysis, decision to publish, or preparation of the manuscript.

Competing Interests: The authors have declared that no competing interests exist.

* E-mail: t.dratsch@mac.com

Introduction

It is hardly possible to imagine today's world without internet-stores and C2C (Consumer to Consumer) platforms, such as Amazon or eBay. Even though online trading provides tremendous advantages in efficiency and accessibility for its users, it has also been characterized as fraught with uncertainty and risk [1–7]. Online transactions are dispersed in space and time [8,9], and communication channels between the market participants are limited [10]. Trust is broadly conceived as a crucial factor in dealing with transaction risks and potentially paralyzing uncertainty [6,11–16]. Flanagin [17] posits: "In essence, C2C commercial transactions entail recurrent initial encounters among strangers who are at significant risk, given the financial and psychological costs of failed transactions and the relative lack of relevant, available information. Thus, engaging in C2C e-commerce requires that at least one party takes a substantial risk and invests trust in someone about whom little is known" (p. 403). As Jarvenpaa et al. [15] hold, trust is an integral part of "any relationship in which the trustor … does not have direct control over the actions of a trustee" (p. 45). It thus can be understood as a form of social capital that enables cooperation under uncertainty [5,18–24].

As Tullberg [25] notes, interpersonal trust comprises trustfulness and trustworthiness. Trustfulness applies to the trustor, who might habitually trust in the good-will and cooperativeness of others, a concept which Rotter [26] already described in an early account as "generalized expectancies for interpersonal trust" (p.443). Trustworthiness applies to the trustee and is fed by his or her reliability that has already been proven in other instances and by the impression that he or she is a "nice guy" who is not expected to defect. As Haley & Fessler [27] put it in more general terms: "..decision-making processes often employ both explicit propositional knowledge and intuitive or affective judgments elicited by tacit cues" (p. 245). These two types of information have already been implemented in C2C platforms by providing reputation scores and seller depictions (photos; avatars). A recent study supports the notion that both factual information about previous cooperative behavior of sellers and seller photos show comparable effects on online trust [1]. Like many preceding studies, this study focused on trust behavior in a Western culture, in this case Germany. However, as cross-cultural research of the past 30 years has shown, culture does exert a great influence on behavior and cognition [28]. Thus, the weight that buyers give to either factual information or visual social cues in online-trust situations might well depend on cultural values and related social evaluation strategies [29,30]. With regard to the progressing globalization of e-commerce markets, identifying cultural patterns in online trust is therefore of major relevance [15,30]. Systematic experimental research, however, is still scarce.

Culture and Online-Trust

Among the cultural dimensions developed by Hofstede [31], two seem of particular importance for the role of trust and the way

it is established in different cultures [32]: Uncertainty Avoidance (UAI) and Individualism/Collectivism (IDV). UAI describes a general tendency to implement rules and to comply with conventions in order to reduce interpersonal uncertainty—thus maybe reducing the need to trust. IDV, on the other hand, distinguishes between cultures assigning higher value to individual responsibility and performance and cultures assigning higher value to collective achievement and interpersonal relationships [33]. IDV in this sense has been related to the preference for two different kinds of trust—namely, cognition-based and affect-based trust [34]. As Kim [30] notes: "Cognition-based trust is built on the knowledge of role performance, whereas affect-based trust is built on the emotional bonds between partners" (p. 443). Chen et al. [35] proposed that cognition-based trust is more important for cooperation in individualistic cultures whereas affect-based trust will be emphasized in collectivistic cultures. In a correlational study, Dakhli [36] divided a group of students into a high collectivism and a high individualism group using the individualism-collectivism scale by Triandis [69]. In addition, Dakhli [36] measured participants affect-based and cognition-based trust using scales developed by McAllister [70]. Participants also rated their willingness to cooperate with another person. In the high collectivism group, affect-based trust was a stronger predictor of the willingness to cooperate with another person than cognition-based trust. In the high individualism group, cognition-based trust was a stronger predictor of the willingness to cooperate with another person than affect-based trust. Also Kim [35] showed that affect-based trust determinants in e-commerce are more positively related to consumer trust in a collectivistic culture, while cognition-based trust determinants were more positively related to consumer trust in an individualistic culture. Given the inter-cultural differences in the relevance of specific trust determinants, we assume that subjects of individualistic vs. collectivistic cultures show a culture-specific bias towards the importance of different trust cues. Indeed, inter-group biases in attention towards information considered as personally important has been shown in a variety of contexts (e.g. [55,56,57]).

Moreover, we recently outlined [1] that although all trust cues aim at fostering trust, different trust cues can directly lead to different types of trust. Reputation scores that provide information about seller cooperativeness stimulate cognition-based trust, while bodily socio-emotional cues, such as seller photos, feed affect-based trust. With respect to buying decisions in an online trust-game, this leads to the assumption that members of individualistic cultures would rely more on factual information, such as records of individuals' cooperative behavior, whereas buyers from collectivistic cultures would rely more on visual social cues, such as a depiction of the seller. Without any doubt, both types of information relate to universals of uncertainty reduction [37], applying to most, if not all, cultures. However, there is also reason to assume that the weight that is put on both factors might be different in individualistic and collectivist cultures [35,36]. The current study aims to explore universals and specificities of trust-building mechanisms, comparing the buying behavior of German and Arab participants in a standard trust game [1,10]. German and Arab cultures were chosen because they are very similar with regard to UAI but differ with regard to IDV [38]. Against this background, we formulated the following hypotheses:

H1: Seller reputation (a) as well as seller depictions (b) will exert significant main effects on online trust in Germans and Arabs.

H2: Reputation will have a higher impact on buying decisions in the individualistic culture (Germans) whereas seller depictions will count more in the collectivistic culture (Arabs), expecting a three-way interaction between culture, reputation, and seller depictions.

To clearly differentiate between factual knowledge and visual social cues, the study used reputation scores as well as seller faces. Factual knowledge about the seller's reliability was framed as automatic system reports indicating the percentage of previous cooperative seller actions instead of user judgements. Seller faces were presented as photo-realistic virtual characters (avatars) instead of photos. The software FaceGen [39] allows to create avatars with standardized facial features and expressions, and to generate faces with different ethnicities from the same basic face models. The effectiveness and validity of this methodology has been demonstrated repeatedly [40–42].

Materials and Methods

Ethics Statement

The ethics committee of the Faculty of Medicine of the University of Cologne (Germany) reviewed the final manuscript of the study and had no objections to publication. All participants were recruited online and participated voluntarily in the study. At the beginning of the study, all participants were informed about the risks and benefits associated with participating in the study. Participants were also informed that they could quit the study at any time by closing the window of their browser. Participants gave their consent to participate by clicking the "Next"-button on the first page of the study. All data were stored and analyzed anonymously.

Study design and experimental setup

The study used a $2\times2\times2$ design, with seller depiction (untrustworthy vs. trustworthy avatar) and reputation (high vs. low cooperativeness in previous transactions) as within-subjects factors and culture (Arab vs. German) as a between-subjects factor. The main dependent variable was the number of purchases made in each condition.

Reputation Scores

We refer to Bente et al. [1] for recommendations of adequate reputation scores on a 5-star-index that is used by major websites (e.g. Amazon). This study identified three stars (the seller shipped 41–60% of past trades) and four stars (the seller shipped 61–80% of past trades) as adequate representatives for moderate distrust and trust, respectively. Both conditions differed significantly on their mean trust ratings, $t(29) = 8.95$, $p < .001$, Cohens's $d = 1.7$. In this study, participants were asked for their trust rating on a 7-point scale with four as the neutral mean. Three stars showed a mean trust rating of $M = 2.90$ ($SD = 1.30$), while four stars were rated with $M = 5.03$ ($SD = 1.22$). Hence, e-commerce users do not perceive three stars as the mean of a five-star scale, instead the neutral anchor is located between three and four stars. Moreover, previous findings showed that three and four stars are used most frequently on five-star scales and that the frequency distribution is remarkably skewed [58], making a mean rating of five stars very unlikely across several users. This also implies that the range of reputation scores is very restricted and hence e-commerce users have to be sensitive to small differences on the scale. In accordance with Bente et al. [1], we consequently used four and three stars as moderately positive and negative reputation scores. The remaining levels of the 5-star-index (1, 2, and 5 stars) were used as filler trials. It has to be noted that in this study reputation was not framed as judgments of other buyers but as real accounts of previous shippings, thus providing an objective measure of cooperativeness. In this context, it is important what Liu [59] recently pointed out:

"Behavioural trustworthiness is not a static or binary value but can be dynamic and indiscrete, evaluating it can be subjective" (p. 61). Hence, a seller who has behaved more reliable in the past does not necessarily continue to do so in the same way. Furthermore, Hardin [20] proposed to distinguish between trust as an attitude and trusting behavior (see also [6,60,61]). According to this differentiation, a higher reputation score can positively influence our trusting beliefs, i.e. the attribution of trustworthiness to the seller. Nevertheless, the reputation might be too low to pass the threshold of trusting behaviour, i.e. to trigger a purchase decision and money transfer. Consequently, the present study intended to clarify, inter alia, whether a difference of one star (three vs. four stars) that was found to indicate a significant difference in the belief in seller trustworthiness elicits significant differences on the buyer's behavioral level. The same logic applies also for seller faces as bodily trust cues.

Seller Avatars

To select trustworthy and untrustworthy avatars, we conducted a pre-study with Arab and German participants. To avoid the influence of stereotypes on decisions, participants in the final trust game were presented only with avatars from their own culture. German participants saw only German avatars, and Arab participants saw only Arab avatars. Therefore, our goal was to select two separate sets of trustworthy and untrustworthy avatars for each culture.

18 male German and 18 male Arab avatars matched for attractiveness and cultural typicality were selected from our database of Arab and German avatars. German avatars were created with the software FaceGen [39], using portrait photos taken from students at the university of Cologne. All participants in the database gave written consent for the use of their photos. Arab avatars were created on the basis of the German avatars using the software's ethnicity transformation tool. Whenever necessary, smiles were eliminated and gaze direction was adjusted to the virtual camera (eye contact).

Attractiveness ratings for all avatars in the database were collected in a pre-study with 42 participants from Germany (38 female, $M_{age} = 23.4$, $SD_{age} = 4.4$) and 31 students from the United Arab Emirates (24 female, $M_{age} = 26.4$, $SD_{age} = 7.9$). Ratings for the cultural typicality and the gender of the avatars were collected in another pre-study with 32 participants from Germany (28 female, $M_{age} = 23.7$, $SD_{age} = 3.9$) and 19 participants from the United Arab Emirates (10 female, $M_{age} = 24.1$, $SD_{age} = 5.7$). Because attractiveness and trustworthiness are strongly associated [43], we only selected Arab and German avatars with moderately positive to moderately negative attractiveness ratings in order to avoid ceiling and floor effects in the final stimulus set. In addition, only avatars that were identified by at least 80% of participants as either German or Arab were selected.

A total of 55 participants took part in the pre-study to identify appropriate avatars for the trust conditions. One Greek participant was excluded from the German sample leaving a sample of 54 participants. 18 participants classified themselves as Arab (9 female, $M_{age} = 20.0$, $SD_{age} = 1.4$), and 36 participants classified themselves as German (26 female, $M_{age} = 36.7$, $SD_{age} = 11.3$).

German and Arab participants rated the trustworthiness of the 36 avatars on a 7-point scale ranging from 1 (very untrustworthy) to 7 (very trustworthy). Both German and Arab participants showed strong intercultural agreement with regard to the trust ratings of the avatars, marked by a strong positive correlations between the trust ratings from the two cultures, $r(40) = .73$, $p < .01$, indicating a strong intercultural agreement on the trustworthiness of the avatars.

Avatars were ranked according to their trust ratings. The six avatars with the highest trust ratings in each culture were selected as the trustworthy avatars; the six avatars with the lowest trust ratings in each culture were selected as the untrustworthy avatars. In both cultures, trustworthy avatars received significantly higher trust ratings than untrustworthy avatars: Trustworthy Arab avatars ($M = 4.32$, $SD = 1.03$) received significantly higher trust ratings from Arab participants than untrustworthy Arab avatars, ($M = 3.07$, $SD = 0.96$), $t(17) = 5.14$, $p < .001$, $d = 1.23$. Trustworthy German avatars ($M = 3.96$, $SD = 0.90$) received significantly higher trust ratings from German participants than untrustworthy German avatars ($M = 3.15$, $SD = 0.89$), $t(35) = 7.83$, $p < .001$, $d = 0.91$. The remaining six avatars from each culture were used as filler trails in the final experiment.

The Online Trust-Game

As in previous studies, we used an adopted version of the standard trust game developed by Bolten [1,10]. In this game, trust situations are framed as sales transactions between a buyer (trustor) and a seller (trustee). Instead of exchanging actual goods, participants are told that they can earn or loose monetary equivalents of goods depending on the action of a seller. Figure 1 shows the payoff matrix of the trust game. Both buyer and seller start each transaction with 35 units (€-Cents). If the buyer decides not to buy (Case 1), both buyer and seller keep their 35 units. If the buyer decides to buy and the seller ships the product (Case 2a), both buyer and seller receive 50 units for the successful trade. If the buyer decides to buy and the seller does not ship the product (Case 2b), the buyer loses his/her 35 units to the seller, who receives 70 units.

Experimental Procedure

The study was conducted as an online experiment using a web application of the trust game. Participants were informed that the goal of the study was to test a new e-commerce platform. They were also informed that the participation was voluntary and that they could abort the study at any time. Participants were led to believe that they were randomly assigned to either the role of the

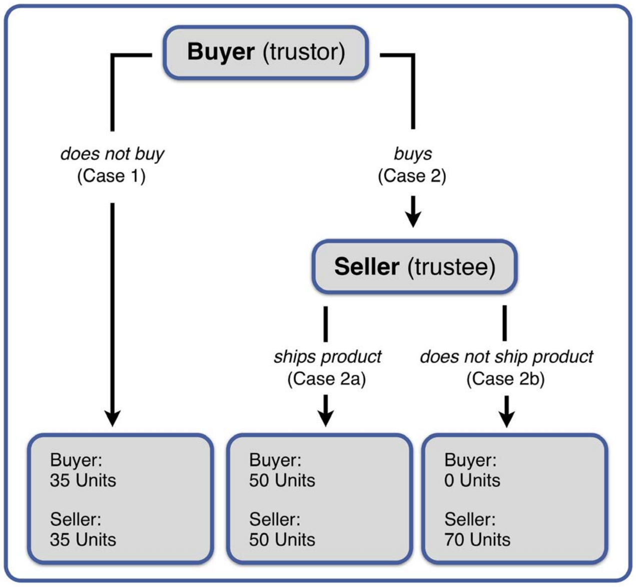

Figure 1. Pay-off matrix for the standard trust game (see Bolton et al., 2004a, p. 188).
doi:10.1371/journal.pone.0098297.g001

seller or the buyer. However, all participants were assigned to the role of the buyer. After that, the generation of the avatars from real photos was explained and illustrated with an example. Hence, although no real photos of sellers were shown participants, knew that each avatar represented a nearly photo-realistic image of the seller. Then the 5-star-reputation score was explained, telling the participants that it reflected the shipment rate of this particular seller in previous transactions.

To increase the reliability of participant's decisions, each participant was presented three times with each of the four combinations of the 2 (moderate untrustworthy vs. moderate trustworthy) x 2 (moderate low reputation vs. moderate high reputation) conditions. Arab participants saw only Arab avatars, and German participants saw only German Avatars. In addition to the twelve trades representing the experimental conditions, six filler trails were included using other reputation scores (1,2, or 5 stars) to mask the repetitive presentation of the 3- and 4-star condition. Two filler trials were presented at the beginning of the experiment as a warm-up phase. All the other trials were displayed in random order. After participants had completed all 18 trades, they answered several demographic questions. Finally, participants were thanked, rewarded, and debriefed. Because there were no real sellers in the game, there were no instances of complete loss. Depending on whether participants did buy or did not buy, they received either 50 or 35 units for each trade. One unit was worth 1 Cent, with the highest possible amount that participants could win being 9 Dollars (18 trades * 0.50 Cent).

Dependent variables

Based on Bolton [44], trust was operationalized through a behavioral measure, counting the positive buying decisions per buyer for each combination of avatar trustworthiness and reputation scores. Buying decisions indicated by a button press on the web interface and were stored as values of either "1" (buy) or "0" (not buy). As each stimulus combination occurred three times per buyer, the value of the dependent variable for each combination of the experimental factors varied between "0" and "3".

Participants

A total of 88 Arab and German participants were recruited via E-Mail using the email lists of the American University of Sharjah and several German universities. Participants were told that they could earn Amazon vouchers worth up to 9 dollars for participating in an e-commerce-study. Four participants were excluded from further analyses. One participant did not belong either to the German or Arab culture. One participant was identified as an outlier with regard to the purchasing behavior because he never bought, reaching a z-score of -3.59 on the purchases variable. Furthermore, two German participants were excluded because they selected the English version of the study and therefore saw Arab instead of German avatars. The final sample consisted of 42 Arab (27 men and 15 women, $M_{age} = 21.29$, $SD = 3.73$) and 42 German (9 men and 33 women, $M_{age} = 27.19$, $SD = 8.95$) participants.

Results

All data were analyzed using IBM SPSS Statistics 21 [45]. First, we recognized an unequal distribution of gender in both cultures, $\chi^2(1,\ N = 84) = 15.75$, $p < .001$. Because some previous studies reported gender differences in trust regarding online shopping [61] and in perceived risk in buying online [62], while other studies did not find evidence for gender differences e-commerce-related trust [63,64], we first checked whether participant's gender had any influence on the results. A $2\times2\times2\times2$ (gender $\times$ culture $\times$ reputation $\times$ seller depictions) mixed ANOVA revealed no significant main effect for gender, $F(1,\ 80) = .31$, $p = .58$, $\eta^2_p = .004$, nor any significant interaction effects, $F < 2.9$. Therefore, gender was dropped as a factor from all further analyses. As one reviewer of an earlier version of the manuscript as well as Smith et al. [65] pointed out, Type-1 errors can propagate across multiple tests within multifactorial ANOVA. The authors hence suggest to adjust the alpha level according to the number of effects tested within a single multifactorial ANOVA. Correspondingly, for the following $2\times2\times2$ ANOVA we only considered p-values below $\alpha_{adj.} = .008$ as statistically significant. On the other hand, however, this Bonferroni-adjusted significance level also leads to a substantial increase in the probability of Type-2 errors, i.e. false negative results as shown in Monte Carlo simulations [65]. Consequently, we applied a very conservative alpha level for statistical significance.

Buying decisions were analyzed in a $2\times2\times2$ (culture $\times$ reputation $\times$ seller depictions) mixed ANOVA. Consistent with Hypothesis 1a, we found a significant main effect for reputation, $F(1,\ 82) = 56.37$ $p < .001$, $\eta^2_p = .41$, showing that buyers across both cultures bought significantly more often from sellers with a high reputation ($M = 2.40$, $SD = .71$) than from sellers with a low reputation ($M = 1.40$, $SD = .99$). As stated in Hypothesis 1b, we also found a significant main effect for seller depictions, $F(1,\ 82) = 17.74$, $p < .001$, $\eta^2_p = .18$, indicating that the trustors in both cultures preferred buying from a seller with a trustworthy avatar ($M = 2.08$, $SD = .65$) than from a seller with an untrustworthy avatar ($M = 1.71$, $SD = .81$). We did not find a main effect for culture, $F(1,\ 82) = 1.56$, $p = .215$, $\eta^2_p = .019$, suggesting that Arabs and Germans did not differ fundamentally in their use of both information types. Contrary to Hypothesis 2, the three-way interaction between reputation, seller depictions, and culture was not significant, $F(1,\ 82) = 0.34$, $p = .854$, $\eta^2_p < .001$.

However, we did find a hint for a two-way interaction between reputation and culture that does not reach the adjusted significance of $\alpha_{adj.} = .008$ but showed a small effect size according to Cohen [66], $F(1,\ 82) = 4.23$, $p = .043$, $\eta^2_p = .049$ (see Figure 2). Pairwise comparisons showed that German participants bought significantly less often when the reputation was low as opposed to Arab participants, $t(82) = -2.03$, $p = .045$, $d = 0.44$. German and Arab participants did not differ significantly when being confronted with a seller with a high reputation score, $t(82) = 0.69$, $p = .493$, $d = 0.14$. It should be noted that this result indicates a possible interaction effect, but it cannot be referred to as a statistically significant effect based on the present data. No significant two-way interactions were found for avatar and culture, $F(1,\ 82) = .30$, $p = .59$, $\eta^2_p = .004$, nor for reputation and avatar, $F(1,\ 82) < .01$, $p = 1.00$, $\eta^2_p < .001$. Although Germans showed stronger negative responses to low reputation than Arabs, this effect did not level out the influence of avatars. As shown in Table 1, trustworthy and untrustworthy avatar depictions produced significant effects in both cultures and under both reputation conditions.

Discussion

We examined the influence of reputation scores and seller avatars on the formation of e-trust in German and Arab participants in an online trust game. Assuming cross-cultural universals in uncertainty reduction [37], we hypothesized that factual information about previous seller behavior (reputation) as well as visual social cues (seller avatars) significantly contribute to

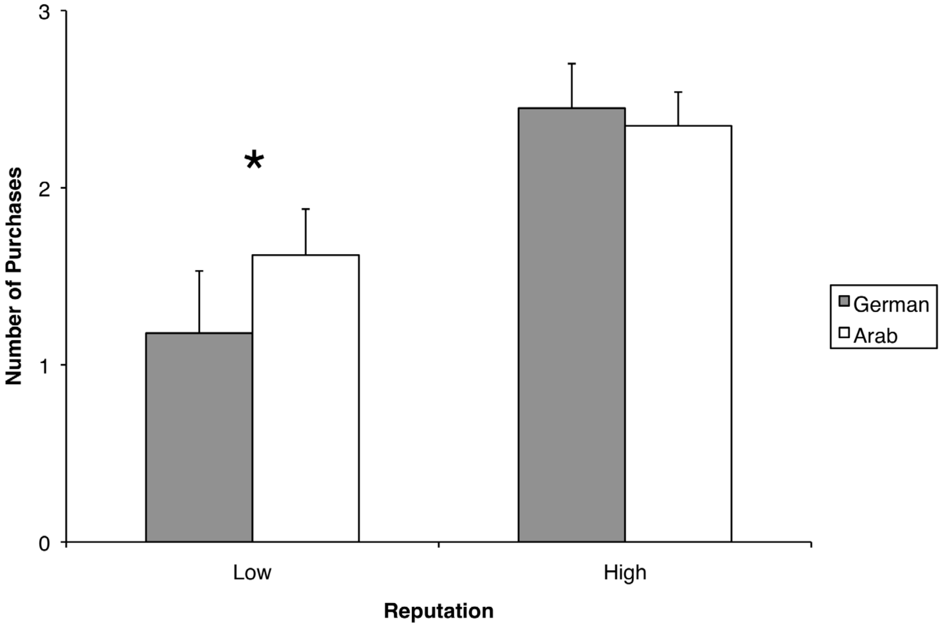

Figure 2. Two-way interaction between reputation and culture. *$p < .05$.
doi:10.1371/journal.pone.0098297.g002

trust (buying decisions under uncertainty) in both cultures. In line with the literature, we further hypothesized that members of an individualistic culture (Germans) would rely more on factual information relevant to cognition-based trust whereas members of a collectivistic culture (Arabs) would be influenced more by relational cues relevant to affect-based trust [30,35,36].

The results support the universality hypothesis, revealing significant main effects for both types of information and for both cultures. Even though this might seem trivial for the effects of factual information, it has to be noted that a moderate difference in trustworthiness in terms of only one star on a five-star scale produces substantial differences on the behavioral level. Apparently, e-commerce users are very sensitive to minimal differences in reputation scores because the range of reputation scores is very restricted in general. Users presumably gathered the implicit knowledge from previous experiences in e-commerce settings that three stars are not perceived as the mean of a five-star scale, as also empirically shown [1], and that three and four stars are used most frequently on five-star scales while the frequency distribution is remarkably skewed [58]. Instead, the neutral anchor is located between three and four stars. Consequently, e-commerce users attribute relevance to marginal differences in reputation scores, leading to behavioral consequences. With respect to Hardin [20], this is a remarkable result as he proposed to distinguish between trust as an attitude and trusting behaviour. A higher reputation score can positively influence our trusting beliefs, i.e. the attribution of trustworthiness to the seller. Nevertheless, the reputation might be too low to pass the threshold of trusting behaviour, i.e. to trigger a purchase decision and money transfer. The present study shows that a difference of one star (three vs. four) is enough to increase money transfer and that this effect is independent of the cultural background of the e-commerce users (German vs. Arab users).

In addition to the effect of reputation scores, it is not self-evident at all that cultures as different as Germany and the United Arab Emirates equally rely on facial cues when it comes to trusting others in first- and single-time interactions—so-called swift trust situations. Swift trust is a presumptive form of trust. If little is known about another person, one categorizes the person automatically into known categories on the basis of the few attributes given [46]. The person is then trusted as other people in that same category [47]. Facial cues can serve as relevant input for categorization. Accordingly, Duarte et al. [67] found that borrowers perceived as less trustworthy are economically and significantly less likely to have their loan requests filled. The interesting fact that participants from both cultures rated the

Table 1. Pairwise t-Test comparisons of trustworthy and untrustworthy avatar conditions, separate for cultures and reputation levels.

		Avatar				
**Culture**	**Reputation**	**Untrustworthy**	**Trustworthy**	***t*(41)**	***p***	**Cohen's *d***
German	High	2.29	2.62	2.47	.018	0.42
	Low	1.02	1.33	2.39	.022	0.37
Arab	High	2.14	2.55	2.72	.010	0.45
	Low	1.40	1.83	2.22	.032	0.35

doi:10.1371/journal.pone.0098297.t001

trustworthiness of the avatars in the pre-study highly similar might indicate a similar cross-cultural swift trust effect or even universal facial features signaling trustworthiness. These possible explanations would have to be explored in future research. As faces do not only signal trustworthiness but also point to the ethnicity of a transaction partner, it would further be interesting to extend the design of this study to cross-cultural transactions in order to explore the relative influence of in-group or out-group membership and face trustworthiness.

The specificity hypothesis claiming that individuals from an individualistic culture (Germans) would put more emphasis on factual information (reputation) whereas those from a collectivistic culture (Arabs) would rely more on interpersonal cues (avatar faces) was only partially supported. The expected three-way interaction between culture, reputation, and avatar did not occur. However, we found a hint for an interaction with small effect size ($p = .043$; $\eta^2_p = .049$) between culture and reputation, indicating that German participants were responding more negatively to low reputation than Arabs ($p = .045$, $d = 0.44$). This result indicates a possible interactional effect, but it has to be assessed with some caution because it did not reach the conservatively adjusted alpha level ($\alpha_{adj.} = .008$) in the present study. However, future studies should test the validity of this effect as it would raise some interesting questions: Because this effect is not symmetrically showing up for positive reputation nor do we find the inverse picture for Arabs, this result pattern cannot be taken as proof for the differential importance of both types of information in the two cultures. The interactional pattern, however, suggests that Germans are more sensitive towards probabilistic indicators of risk. Information revealing that the seller shipped only in 41 to 60% of previous trials led to a significant drop in buying decisions below chance level only in the German sample. Even though this effect does not support predictions based on Chen [35] and Kim [30], it is in line with results by Dakhli [36] on trust behavior and the IDV dimension of culture. They found that, beyond the expected cultural differences in the relevance attached to either cognition- or affect based trust, the relationship between trust (trusting attitudes) and cooperative (trusting) behavior in general is stronger in individualistic cultures. Furthermore, such an interactional effect would raise the question whether there is a cultural bias towards the "negativity effect" [48] (as well established in social psychology [49–51]) or "risk-aversion" [52]. For instance, Standifird [53] found asymmetric effects in favor of negative reputation for final bids in eBay auctions. As in most studies on online-trust, he only investigated participants from a Western culture. The possibility of a cultural negativity bias in online-trust should therefore be explored in more detail in future studies. Alternatively, a more negative behavioral response of Germans in the case of low reputation scores might lead to the assumption that the relative reliance on statistical information might depend on statistical training or even experience in e-trading more than anything else including individualism or collectivism. Perhaps German participants use e-trading websites more than Arabs do.

In contrast to recent studies using seller photos, effect sizes for avatar depictions were smaller in the current study than those for reputation scores [1]. Accordingly, Rezlescu [68] found exactly the same pattern, with investment decisions greatly influenced by reputation and to a lesser extent by faces. They also used Facegen faces as in the present study. This result might be interpreted as a kind of "digital trust discount"-effect, which might generally apply to virtual characters. Although the facial features of virtual characters were able to trigger evaluation processes relevant to trust, which is consistent with recent findings from cognitive neuroscience [42], the artificial nature of the avatar faces could, to a certain degree, have inhibited the strong affective responses found for portrait photos. This has far reaching implications for the use of avatars in research as well as in shared virtual realty applications, such as virtual shops or market places. It would be most relevant to further explore how far real life principles of person perception and impression formation apply to such settings [54] and which features characterize trustworthy and effective avatars in the virtual worlds and future immersive online markets. On the other hand, it has to be noted that the FaceGen avatars [39] appear very human-like as real portrait photos are applied on a polygonal head model. Importantly, the generation process of the avatars from real photos was explained to participants and illustrated with an example. Hence, although we did not show real photos of sellers participants knew that each avatar represented a nearly photo-realistic image of the seller. This fact is noteworthy as an increasing difference in the morphological concordance between the seller's real face and the avatar's face would change the question at a certain point to "Is this person using this seemingly trustworthy (or not too trustworthy) avatar really trustworthy?". Then, the avatar trustworthiness might have nothing to do with actual perceived trustworthiness. Consequently, given the possibility to control for many facial features when using avatars in contrast to real portrait photos, researchers should consider the potential impact of avatar quality on the interpretation of results.

Acknowledgments

We wish to thank Eva-Louisa Lamerz, Christian Remke and Eva Röttger for their support in the realization of this study.

Author Contributions

Conceived and designed the experiments: GB TD TH OB AA. Performed the experiments: TD TH OB. Analyzed the data: TD TH OB. Contributed reagents/materials/analysis tools: GB TD TH OB. Wrote the paper: GB TD KK TH OB. Revised the manuscript: KK TD.

References

1. Bente G, Baptist O, Leuschner H (2012) To buy or not to buy: Influence of seller photos and reputation on buyer trust and purchase behavior. International Journal of Human-Computer Studies 70: 1–13.
2. Egger FN (2001) Affective design of e-commerce user interfaces: How to maximise perceived trustworthiness. Proceedings of the international conference on affective human factors design: 317–324.
3. Grabner-Kräuter S, Kaluscha EA (2003) Empirical research in on-line trust: a review and critical assessment. International Journal of Human-Computer Studies 58: 783–812.
4. Metzger J (2006) Effects of Site, Vendor, and Consumer Characteristics on Web Site Trust and Disclosure. Communication Research 33: 155–179.
5. Riegelsberger J, Sasse MA, McCarthy JD (2005) The mechanics of trust: A framework for research and design. International Journal of Human-Computer Studies 62: 381–422.
6. Riegelsberger J, Sasse MA, McCarthy JD (2007) Trust in mediated interactions. In: McKenna K, Postmes T, Reips U, Joinson AN, editors. The Oxford handbook of Internet psychology. Oxford: Oxford University Press. pp. 53–70.
7. Tan Y-H, Thoen W (2001) Toward a generic model of trust for electronic commerce. International Journal of Electronic Commerce 5: 61–74.
8. Brynjolfsson E, Smith MD (2000) Frictionless commerce? A comparison of Internet and conventional retailers. Management Science 46: 563–585.
9. Riegelsberger J, Angela Sasse M (2002) Trustbuilders and trustbusters. International Federation for Information Processing 74: 17–30.
10. Bolton GE, Katok E, Ockenfels A (2004) How Effective Are Electronic Reputation Mechanisms? An Experimental Investigation. Management Science 50: 1587–1602.
11. Corritore C, Kracher B, Wiedenbeck S (2001) Trust in the online environment. HCI International 1: 1548–1552.

12. Fogg BJ, Marshall J, Laraki O, Osipovich A, Varma C, et al (2001) What makes Web sites credible?: a report on a large quantitative study. Proceedings of the SIGCHI conference on Human factors in computing systems: 61–68.
13. Gefen D (2000) E-commerce: the role of familiarity and trust. Omega 28: 725–737.
14. Gefen D, Karahanna E, Straub DW (2003) Trust and TAM in online shopping: an integrated model. MIS quarterly: 51–90.
15. Jarvenpaa SL, Tractinsky N, Vitale M (2000) Consumer Trust in an Internet Store. Information Technology and Management 5: 45–71.
16. Wang YD, Emurian HH (2005) An overview of online trust: Concepts, elements, and implications. Computers in Human Behavior 21: 105–125.
17. Flanagin J (2007) Commercial markets as communication markets: uncertainty reduction through mediated information exchange in online auctions. New Media & Society 9: 401–423.
18. Corritore CL, Kracher B, Wiedenbeck S (2003) On-line trust: concepts, evolving themes, a model. International Journal of Human-Computer Studies 58: 737–758.
19. Glaeser EL, Laibson DI, Scheinkman JA, Soutter CL (2000) Measuring trust. The Quarterly Journal of Economics 115: 811–846.
20. Hardin R (2001) Conceptions and explanations of trust. In: Cook KS, editors. Trust in society. New York: Russell Sage Foundation. pp. 3–39.
21. Lewis JD, Weigert A (1985) Trust as a social reality. Social forces 63: 967–985.
22. Misztal BA (1996) Trust in modern societies: The search for the bases of social order. New York: Polity Press Cambridge.
23. Putnam RD (1995) Bowling alone: America's declining social capital. Journal of democracy 6: 65–78.
24. Uslaner EM (2000) Social capital and the net. Communications of the ACM 43: 60–64.
25. Tullberg J (2008) Trust—The importance of trustfulness versus trustworthiness. The Journal of Socio-Economics 37: 2059–2071.
26. Rotter JB (1971) Generalized expectancies for interpersonal trust. American psychologist 26: 443–452.
27. Haley KJ, Fessler DMT (2005) Nobody's watching? Subtle cues affect generosity in an anonymous economic game. Evolution and Human Behavior 26: 245–256.
28. Markus HR, Kitayama S (1991) Culture and the self: Implications for cognition, motivation, and emotion. Psychol Rev 98: 224–253.
29. Gheorghiu MA, Vignoles VL, Smith PB (2009) Beyond the United States and Japan: testing Yamagishi's emancipation theory of trust across 31 nations. Social Psychology Quarterly 72: 365–383.
30. Kim DJ (2005) Cognition-based versus affect-based trust determinants in e-commerce: a cross-cultural comparison study. Proceedings of the 26th International Conference on Information Systems: 11–14.
31. Hofstede G (2003) Cultures Consequences. Comparing values, behaviors, institutions and organizations across nations. Thousand Oaks, CA: Sage Publications.
32. Kao DT (2009) The impact of transaction trust on consumers' intentions to adopt m-commerce: a cross-cultural investigation. Cyberpsychol Behav 12: 225–229.
33. Huff L, Kelley L (2003) Levels of organizational trust in individualist versus collectivist societies: A seven-nation study. Organization Science 14: 81–90.
34. McAllister DJ (1995) Affect-and cognition-based trust as foundations for interpersonal cooperation in organizations. Academy of management journal: 24–59.
35. Chen CC, Chen X-P, Meindl JR (1998) How can cooperation be fostered? The cultural effects of individualism-collectivism. Academy of Management Review: 285–304.
36. Dakhli M (2009) Investigating the Effects of Individualism-Collectivism on Trust and Cooperation. Psychology Journal 6: 90–99.
37. Berger CR, Calabrese RJ (1975) Some explorations in initial interaction and beyond: Toward a developmental theory of interpersonal communication. Human Communication Research 1: 99–112.
38. Hofstede G, Hofstede GJ, Minkov M (2010) Cultures and organisations: Software of the mind. New York: McGraw-Hill.
39. (2012) FaceGen. Singular Inversions Inc.
40. Bente G, Dratsch T, Rehbach S, Reyl M, Lushaj B (2014) Do you trust my avatar? Influence of seller avatars and reputation on trust in online transactions. LNCS 8527: 461–471.
41. Krämer K, Bente G, Luo S, Pfeiffer P, Han S, Vogeley K Influence of Ethnic Group-Membership and Gaze Direction on the Perception of Emotions. A Cross-Cultural Study between Germany and China. PLoS ONE 8(6): e66335.
42. Todorov A (2008) Evaluating faces on trustworthiness: an extension of systems for recognition of emotions signaling approach/avoidance behaviors. In: Kingstone A, editors. Ann N Y Acad Sci. United States. pp. 208–224.
43. Wilson K, Eckel C (2006) Judging a Book by its Cover: Beauty and Expectations in the Trust Game. Political Research Quarterly 59: 189–202.
44. Bolton GE, Katok E, Ockenfels A (2004) Trust among Internet traders: A behavioral economics approach. Analyse und Kritik 26: 185–202.
45. (2012) IBM SPSS Statistics 21. IBM.
46. Meyerson D, Weick KE, Kramer RM (1996) Swift trust and temporary groups. In: Kramer RM, Tyler TR, editors. Trust in organizations: Frontiers of theory and research. Thousand Oaks, CA: Sage. pp. 166–195.
47. McKnight DH, Cummings LL, Chervany NL (1998) Initial trust formation in new organizational relationships. Academy of Management review: 473–490.
48. Kellermann K (1989) The negativity effect in interaction it's all in your point of view. Human Communication Research 16: 147–183.
49. Anderson NH (1965) Averaging versus adding as a stimulus-combination rule in impression formation. Journal of Experimental Psychology 70: 394–400.
50. Birnbaum MH (1972) Morality judgments: Tests of an averaging model. Journal of Experimental Psychology 93: 35–42.
51. Fiske ST (1980) Attention and weight in person perception: The impact of negative and extreme behavior. Journal of Personality and Social Psychology 38: 889–906.
52. Kahneman D, Tversky A (1979) Prospect theory: An analysis of decision under risk. Econometrica 47: 263–292.
53. Standifird S (2001) Reputation and e-commerce: eBay auctions and the asymmetrical impact of positive and negative ratings. Journal of Management 27: 279–295.
54. Yee N, Bailenson JN, Urbanek M, Chang F, Merget D (2007) The unbearable likeness of being digital: the persistence of nonverbal social norms in online virtual environments. CyberPsychology & Behavior 10: 115–121.
55. van Reekum CM, Scherer KR (1997) Levels of processing in emotion-antecedent appraisal. In: G. Matthews, editor. Cognitive science perspectives on personality and emotion. Amsterdam: Elsevier. pp. 259–330.
56. Rosser BA, Moss T, Rumsey N (2010) Attentional and interpretative biases in appearance concern: An investigation of biases in appearance-related information processing. Body Image 7: 251–254.
57. Derryberry D, Reed MA (1994) Temperament and attention: Orienting toward and away from positive and negative signals. Journal of Personality and Social Psychology 66: 1128–1139.
58. Tan TF, Netessine S (2009) Is Tom Cruise threatened? Using Netflix Prize data to examine the long tail of electronic commerce. University of Pennsylvania, Philadelphia: Wharton Business School. pp. 1–37.
59. Liu WW (2011) Trust Management and Accountability for Internet Security. The Florida State University, Florida: *Electronic Theses, Treatises and Dissertations*. Paper 1105.
60. McKnight DH, Chervany NL (2001) What trust means in e-commerce customer relationships: an interdisciplinary conceptual typology. International Journal of Electronic Commerce 6: 35–59.
61. Yang SH, Hung WC, Sung K, Farn CK (2006) Investigating initial trust towards e-tailers from the elaboration likelihood model perspective. Psychology & Marketing 29: 420–455.
62. Rodgers S, Harris MA (2003) Gender and e-commerce: an exploratory study. Journal of Advertising Research 43: 322–329.
63. Garbarino E, Strahilevitz M (2004) Gender differences in the perceived risk of buying online and the effects of receiving a site recommendation. Journal of Business Research 57: 768–775.
64. Kolsaker A, Payne C (2002) Engendering trust in e-commerce: a study of gender-based concerns. Marketing Intelligence & Planning 20: 206–214.
65. Smith RA, Levine TR, Lachlan KA, Fediuk TA (2002) The high cost of complexity in experimental design and data analysis: Type I and type II error rates in multiway ANOVA. Human Communication Research, 28: 515–530.
66. Cohen J (1988) Statistical Power Analysis for the Behavioral Sciences (2nd Edn.). Hillsdale, New Jersey: L. Erlbaum.
67. Duarte J, Siegel S, Young L (2012) Trust and credit: the role of appearance in peer-to-peer lending. Review of Financial Studies 25: 2455–2484.
68. Rezlescu C, Duchaine B, Olivola CY, Chater N (2012) Unfakeable facial configurations affect strategic choices in trust games with or without information about past behavior. PloS ONE 7: e34293.
69. Triandis HC (1995) Individualism and collectivism. Boulder, CO: Westview.
70. McAllister DJ (1995) Affect- and cognition-based trust as foundation for interpersonal cooperation in organizations. Academy of Management Journal 38: 24–59.
